# Alleviation of taurine on liver injury of type 2 diabetic rats by improving antioxidant and anti-inflammatory capacity

**DOI:** 10.1016/j.heliyon.2024.e28400

**Published:** 2024-03-19

**Authors:** Guangyi Ouyang, Nannan Wang, Jihang Tong, Wenke Sun, Jiancheng Yang, Gaofeng Wu

**Affiliations:** Liaoning Provincial Key Laboratory of Zoonosis, College of Animal Science & Veterinary Medicine, Shenyang Agricultural University, Shenyang, 110866, China

**Keywords:** Taurine, Oxidative stress, Inflammatory response, Liver injury, Type 2 diabetic rats

## Abstract

Type 2 diabetes mellitus (T2DM) is a serious metabolic disease characterized by insulin resistance and reduced insulin production, which causes abnormally elevated blood glucose. It has been reported that T2DM can enhance oxidative stress and inflammatory responses, and stimulate a variety of complications including liver injury. Studies have shown that taurine has antioxidant and anti-inflammatory effects that can not only ameliorate diabetes but also alleviate liver injury caused by various diseases. However, its effect on liver injury in T2DM is not clear. In our study, a high-fat diet and intraperitoneal injection of streptozotocin (STZ) was used to induce liver injury in T2DM rats, and taurine was given as a treatment. Through the use of HE staining on paraffin sections, ELISA, and qRT-PCR, the effects of taurine on liver pathological alterations, antioxidant capacity, and inflammatory response were investigated. We found that: hepatic transaminase levels of rats were reduced significantly following taurine administration; histopathological observations revealed that the morphology of rat hepatocytes was close to normal, and the number of inflammatory cells around liver vessels was significantly reduced; antioxidant-related indicators were significantly increased, including SOD, CAT, GSH-Px and T-AOC, while related factors of the Nrf2 signalling pathway and its downstream HO-1, NQO1 and γ-GCS were significantly increased; the expression of the JAK2-STAT1 signalling pathway, TLR4/NF-κB signalling pathway and NLRP3 inflammatory vesicle-related factors were significantly reduced. Our results suggest taurine can alleviate T2DM-induced liver injury by improving the antioxidant capacity of the liver and inhibiting macrophage M1-type polarization and the inflammatory response mediated thereby.

## 1 Introduction

Hyperglycemia is a symptom of diabetes mellitus (DM). DM is often accompanied by many complications, including chronic injury to the liver, kidney, heart and other tissues, which has become a serious hazard to human health. Data from the WHO show that the number of adult diabetic patients has roughly tripled since 1980 and reached 422 million. It is estimated that there will be 693 million DM patients by 2045 worldwide, of whom type 2 DM (T2DM) patients account for 90–95% of the total [1]. The causes of T2DM include peripheral insulin resistance (IR), impaired regulation of hepatic glucose production, and decreased β-cell function, which ultimately lead to the progressive failure of pancreatic β-cells. It has been reported that IR caused by the occurrence of T2DM can lead to the aggravation of hyperglycemia and induce various forms of liver injury, ultimately, fatty liver, cirrhosis, and liver cancer result from this condition [2,3]. The main target organ of IR is liver and it is susceptible to hyperglycemia, resulting in metabolic abnormalities and dysfunction and leading to T2DM liver injury, which further exacerbates IR and forms a vicious cycle [4]. Oxidative stress and inflammation are closely related to T2DM [5]. Inflammation, oxidative stress, and peripheral IR are the key pathological mechanisms leading to liver injury [6,7]. Increasing levels of glucose in the peripheral blood and lipid metabolites accumulate in the liver, causing oxidative stress and inflammation, resulting a large amount of reactive oxygen species (ROS) and inflammatory cytokines are released, then causing liver injury [8,9]. By stimulating the polarization of Kupffer cells (KCs) in liver, a large amount of inflammatory cytokines can increase the infiltration of macrophages and the release of proinflammatory cytokines and activate the production of inflammatory cytokines and ROS, further causing liver IR and the inflammatory response, thereby exacerbating T2DM liver injury [10,11].

Taurine, one of the natural antioxidants, is a simple structure, widely present in the body and can be found in the heart, liver, brain, retina, muscle and other tissues or organs [12,13]. There are many biological functions it has, including antioxidation, anti-inflammation, osmotic regulation, calcium homeostasis, bile salt formation, and regulating central nervous system functions [14]. In diseases with increased oxidative stress and inflammation, such as diabetes, obesity, and metabolic syndrome, taurine may be a conditional and essential amino acid [15]. Oxidative stress is reduced by taurine, which is a scavenger of oxygen free radicals and a modifier of the antioxidant system [16,17]. Studies have found that taurine can prevent lipopolysaccharide-induced liver injury in weaned piglets by inhibiting apoptosis. In addition, taurine can alleviate aflatoxin B1-induced liver injury in rats by reducing oxidative stress and regulating mitochondrial-mediated apoptosis; taurine supplementation in the diet can significantly improve tunicamycin-induced liver injury in mice, and it can reduce liver injury in rats through anti-inflammatory and anti-apoptotic pathways. Numerous investigations have revealed that taurine can enhance antioxidant enzymes (for example, SOD (superoxide dismutase), CAT (catalase), GSH-Px (glutathione peroxidase)), improve total T-AOC (antioxidant capacity), and maintain redox levels; taurine possesses the potential to initiate the Nrf2 pathway, consequently boosting the operation of antioxidant enzymes downstream, thereby mitigating the detrimental effects of oxidative stress [16,[18], [19], [20]]. In addition, taurine can reduce the levels of inflammatory cytokines such as IL-1β, iNOS, and IL-8 and downregulate the Toll-like receptor 4 (TLR4)/NF-κB signalling pathway and its downstream inflammatory genes to alleviate inflammation and oxidative injury [[21], [22], [23]]. Hence, our conjecture entails that taurine holds the potential to mitigate T2DM-triggered liver injury through enhancing the antioxidative prowess and curbing the inflammatory rejoinder. In this study, a liver injury model of T2DM rats was induced by a high-fat diet and intraperitoneal injection of streptozotocin (STZ), and taurine was given for treatment, HE staining of paraffin sections, ELISA, qRT-PCR and other methods were used to detect taurine's effects on liver pathology, antioxidant capacity, macrophage M1-type polarization and inflammatory response mediated thereby. The regulating influence of taurine on liver injury in the occurrence phase of T2DM and its potential mechanism were elucidated, it provides a theoretical basis for the application of taurine in the prevention and treatment of T2DM liver injury.

## 2 Materials and methods

### 2.1 Experimental design

Eight-week-old male Sprague Dawley (SD) rats, weighing between 180 and 200 g, were housed at room temperature with approximately 50% humidity and were provided with light for 12 h and darkness for 12 h each day to simulate alternating day and night. The SD rats were acclimated for one week, after which the rats were randomly divided into three groups (10 rats in each group) according to body weight (±20 g): normal group (Con group), diabetes model group (DM group), and treatment group (DM +T group). The Con group was always given GB D12450B standard chow; during the first to eighth week, the DM and DM +T groups were given GB D12492 high-fat chow, and after 8 weeks, in addition to the normal group, the T2DM rat model was induced by intraperitoneal injection of STZ at a dose of 30 mg/kg. After successful modelling, according to the results of previous studies, our treatment group was treated with 2 % taurine drinking water, and the remaining treatment was unchanged [24]. Taurine (purity>99%) (Huaheng, Hebei, China). After 11 weeks of treatment, all rats were sacrificed by bloodletting after intraperitoneal injection of urethane anesthetic (1.2 g/kg), and measured body weight before execution. We collected blood samples and conducted centrifugation to obtain serum. Furthermore, we gathered liver tissues to facilitate subsequent experiments. All laboratory animals were cultivated and worked in Shenyang Agricultural University. All procedures for animal experiments have been reviewed and approved by the Animal Care and Use Committee of Shenyang Agricultural University (2019080501).

### 2.2 Blood glucose assessment

With an ACCU-CHEK blood glucose meter, blood glucose levels were measured by collecting blood from the rats' tails.

### 2.3 Histopathological detection

The rat liver tissue blocks were fixed in 4% paraformaldehyde, removed from the specimen bottles, and prepared as follows: rinsing, dehydration and transparency, wax immersion and embedding, and sectioning and gluing. The operation was carried out according to the instructions of HE staining kit. After staining, neutral gum was used to seal the sections, and observation was performed through microscope after the sections were completely dry.

### 2.4 Determination of serum biochemical indices

The rats serum was detected according to the instructions of ALT assay kit (cat. No. C009-2-1) and AST assay kit (cat. No. C010-2-1) from the Nanjing Jiancheng Institute of Biological Engineering.

### 2.5 Determination of liver antioxidant indices

First, BCA was used to determine the total protein content of liver homogenate, then the liver was detected according to the instructions of SOD assay kit (cat. No. A001-3-2), CAT assay kit (cat. No. A007-1-1), GSH-Px assay kit (cat. No. A005-1-2), T-AOC assay kit (cat. No. A015-2-1), MDA assay kit (cat. No. A003-1-2) from the Nanjing Jiancheng Institute of Biological Engineering.

### 2.6 Detection of inflammatory factors in liver tissue

The contents of IL-1β and interferon-γ (IFN-γ) in the liver tissue of rats in each group were detected by the multifactor detection method. The ELISA kit to determine contents of iNOS (cat. No. E-EL-R0520), NO (cat. No. E-BC-K035-S), IL-8, and IL-18(cat. No. E-EL-R0567) in the liver tissue of rats (Elabscience, China).

### 2.7 Real-time PCR analysis

After the extraction of liver RNA using TRIzol reagent, the purity of RNA was assessed using a microplate reader. The OD260/OD280 ratio was measured after adjusting to zero with double distilled water. The ratio was in the range of 1.8–2.0, indicating that the purity of the RNA was good and met the experimental requirements. Then, RNA was converted into cDNA using a reverse transcription kit following the manufacturer's instructions.

By real-time quantitative polymerase chain reaction, mRNA expression levels of related factors were measure. The full sequences of the genes were searched in GenBank on NCBI, and Primer Premier5 was used for designing the primer sequences which showed in Table 1. After the primer specificity was verified to be good, SYBR Green II was used for qRT-PCR, and used β-actin as an internal reference, target gene expression was normalized. The mRNA levels were analysed by the 2 ^−ΔΔCT^ method.

**Table 1:** Primer sequences used for fluorescence quantitative real-time PCR.

Gene	Primer sequences	GenBank Accession number	Product size(bp)
Nrf2	F: CTCCTTAGACTCAAATCCCACCTT R: GGACAGATCACAAGCCCTCAAT	NM_001399173.1	164
HO-1	F: ATGAGGAACTTTCAGAAGGGTC R: GTGGGGCATAGACTGGGTT	XM_039097470.1	130
NQO1	F:TCAAGAGGAGCAGAAAAAGAACAAG R: CTGAAAGCAAGCCAGGCAAAC	NM_017000.3	162
γ-GCS	F: GAGCGAGATGCCGTCTTACA R: TTGCTACACCCATCCACCAC	NM_012815.2	86
F4/80	F: CAGCTGTCTTCCCGACTTTC R: TAATCAAGATTCCGGCCTTG	XM_039083468.1	156
CD68	F: TTGGATTCAAACAGGACCGA R: TTGCTGGAGAAAGAACTATGC	NM_001031638.1	195
LBP	F: TGACTACAGTTTGGTGGCGG R: TTGGTGTTCAGCCGGATGTT	NM_017208.2	273
TLR4	F: CAGAATGAGGACTGGGTGAG R: AGGACAATGAAGATGATGCC	NM_019178.2	266
MyD88	F: TATACCAACCCTTGCACCAAGTC R: TCAGGCTCCAAGTCAGCTCATC	NM_198130.2	121
NF-κB	F: GACGACACCTCTACACATAGCA R: CCTCATCTTCTCCAGCCTTCTC	NM_001276711.2	146
AP-1	F: TAGCCAACTTTATCCCCACG R: CTCTACTTTGCCCCTTCTGC	NM_022197.2	221
IRF-3	F: GCAGACATTCCCCACGATAC R: GCAAGTCCACGGTTTTCAGT	NM_001006969.1	171
NLRP3	F: GCTCAAAACCAACCAGAA R: GCAGAAGTCCCTCACAGA	NM_001191642.1	92
Caspase-1	F: GAGAGAAACAAGGAGTGGTG R: AAGAGCAGAAAGCAATAAAATC	NM_012762.3	135
Caspase-11	F: ACAACCACCCTGATAAACCA R: CTGCCTGAGTCCACATTAAGAA	NM_053736.2	238
JAK2	F: GGAATGGCCTGCCTTACAATG R: TGGCTCTATCTGCTTCACAGAAT	NM_031514.1	108
STAT1	F: GGCGAAGAGCGACCAGAAAC R: AGCTGATCCAGGCAGGCATT	NM_032612.3	216
β-actin	F: GAGAGGGAAATCGTGCGTGACA R: CGATAGTGACCTGACCGTCA	NM_031144.3	136

### 2.8 Statistical analysis

The obtained data were analysed by SPSS23.0 software. Differences between groups were analysed using one-way ANOVA. The measurement data are expressed as the mean ± SEM. p < 0.05 was statistically significant.

## 3 Results

### 3.1 Blood glucose assessment

**Fig. 1:**
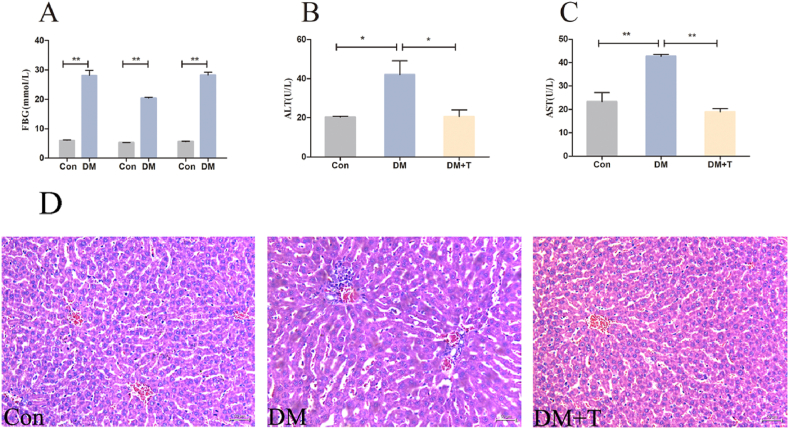
Detection of blood glucose and liver function in rats. (**A**) The results of three fasting blood glucose tests in T2DM rats. (**B**) The serum ALT level in T2DM rats. (**C**) The serum AST level in T2DM rats. (**D**) The analysis of liver tissue sections in T2DM rats (H.E 200x). The results were expressed by the mean ± SEM. *p < 0.05 and **p < 0.01.

After the rats were intraperitoneally injected with STZ, we examined their blood glucose levels, and the results are shown in Fig. 1A. The blood glucose levels of rats in the DM model group were detected three times and were significantly increased (p < 0.01) compared with those in the normal group and were higher than 11.1 mmol/L for more than two weeks. Therefore, we believe that the model is established successfully.

### 3.2 Assessment of liver function and liver injury

ALT and AST, which are indicators of liver function, are released into the blood when hepatocytes are damaged due to increased cell membrane permeability, resulting in increased levels of ALT and AST in the serum. The levels of ALT and AST in the serum of the test rats are shown in Fig. 1B and C, compared to the control group, the diabetes model group exhibited notably elevated serum ALT and AST levels (p < 0.05), which were significantly reduced following taurine treatment (p < 0.01).

In order to further observe the histopathological changes in the liver, we performed HE staining. Fig. 1D shows the results of H.E staining of rat liver sections. The normal group had clear hepatocyte structure and no inflammatory cell aggregation around liver vessels, while the diabetes model group had disordered hepatocyte morphology and structure and a large amount of inflammatory cell accumulation around liver vessels. After the addition of taurine treatment, the hepatocyte morphology was close to normal, and the number of inflammatory cells around liver vessels was significantly reduced.

### 3.3 Effect of taurine on the antioxidant capacity of the liver of T2DM rats

**Fig. 2:**
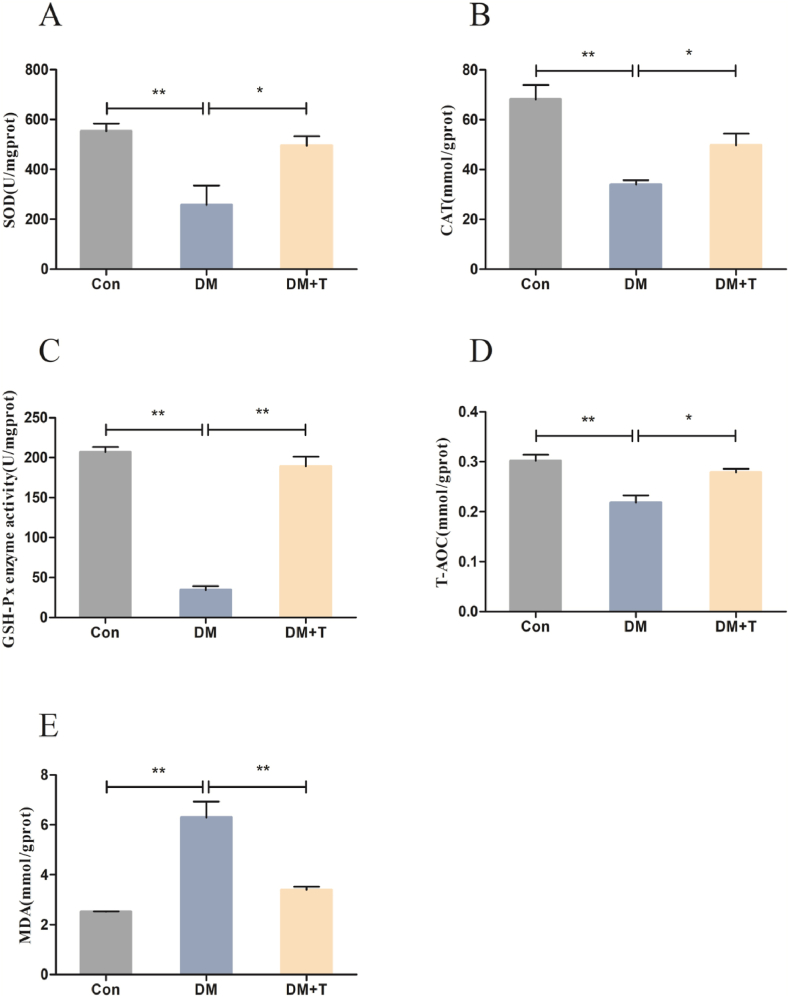
Results of the liver antioxidant capacity assay of T2DM rats. (**A**) The liver SOD levels of T2DM rats. (**B**) The liver CAT levels of T2DM rats. (**C**) The liver GCS- Px levels of T2DM rats. (**D**) The liver T-AOC levels of T2DM rats. (**E**) Results of liver MDA content of T2DM rats. The results were expressed by the mean ± SEM. *p < 0.05 and **p < 0.01.

Fig. 2 presents the data on antioxidant indices in rat liver. As shown in Fig. 2A–D, compared to the normal group, the diabete model group exhibited significantly decreased levels of SOD, CAT, GSH-Px, and T-AOC (p < 0.01), and SOD, CAT, GSH-Px and T-AOC contents were significantly increased after treatment with taurine (p < 0.05). Fig. 2E presents the findings regarding the liver MDA content in rats. The results indicated a significant increase in MDA content in the diabetes model group compared to the normal group (p < 0.01). However, upon treatment with taurine, MDA content showed a marked decrease (p < 0.01).

**Fig. 3:**
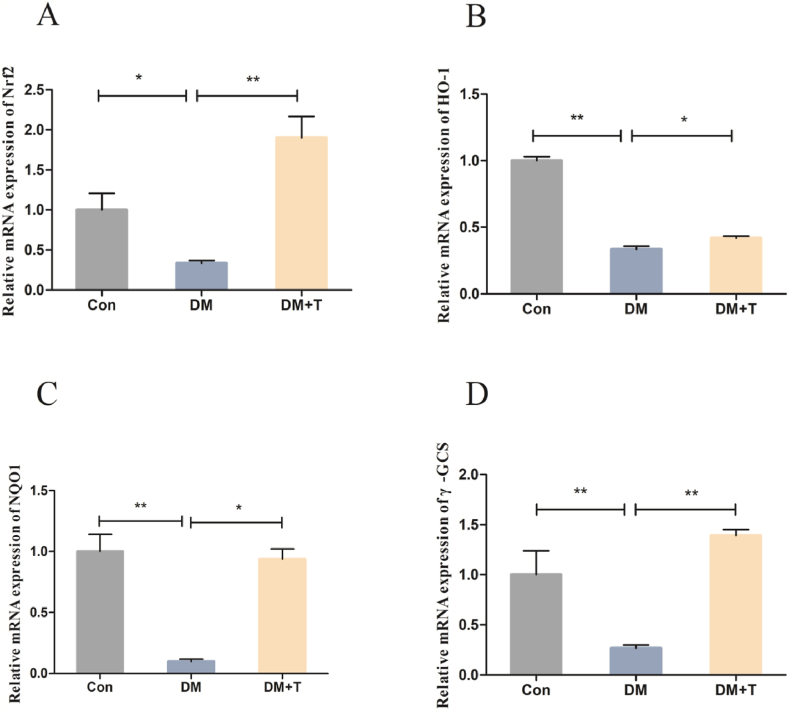
Results of the Nrf2 antioxidant pathway indices of the liver of T2DM rats. (**A**) The Nrf2 mRNA expression of T2DM rats. (**B**) The HO-1 mRNA expression of T2DM rats. (**C**) The NQO1 mRNA expression of T2DM rats. (**D**) The γ-GCS mRNA expression of T2DM rats. The results were expressed by the mean ± SEM. *p < 0.05 and **p < 0.01.

To better understand how taurine increases the antioxidant capacity of T2DM rats' livers, we examined indices related to the Nrf2 antioxidant pathway, and the results are shown in Fig. 3. As shown in Fig. 3A–D, in the diabetes model group, the mRNA expression levels of Nrf2, HO-1, NQO1, and γ-GCS showed a significant decrease when compared to those in the normal group (p < 0.05). However, upon administering taurine treatment, a substantial increase was observed in the expression levels of these genes (p < 0.05).

### 3.4 Effect of taurine on the inflammatory response in the liver of T2DM rats

**Fig. 4:**
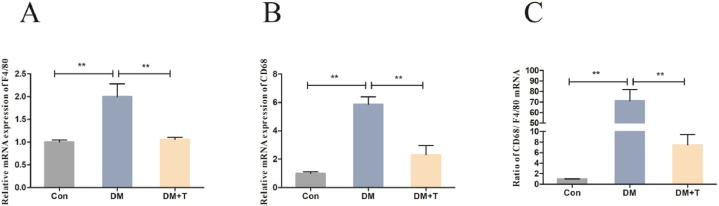
Results of liver KCs marker assay of T2DM rats. (**A**) The F4/80 mRNA expression of T2DM rats. (**B**) The CD68 mRNA expression of T2DM rats. (**C**) Ratio of CD68 to F4/80. The results were expressed by the mean ± SEM. *p < 0.05 and **p < 0.01.

When liver macrophages are subjected to external stimuli, they polarize towards the M1 type and release large amounts of proinflammatory cytokines. We examined M1-type KCs markers, and the results were showed in Fig. 4A–C. In comparison to the control group, the levels of F4/80, CD68, and CD68/(F4/80) mRNA were considerably elevated (p < 0.01) in the diabetes model group. Conversely, upon the administration of taurine treatment, the mRNA expression levels of F4/80, CD68, and CD68/(F4/80) experienced a significant reduction (p < 0.01).

### 3.5 Effect of taurine on the levels of inflammatory response-related factors in the liver of T2DM rats

**Fig. 5:**
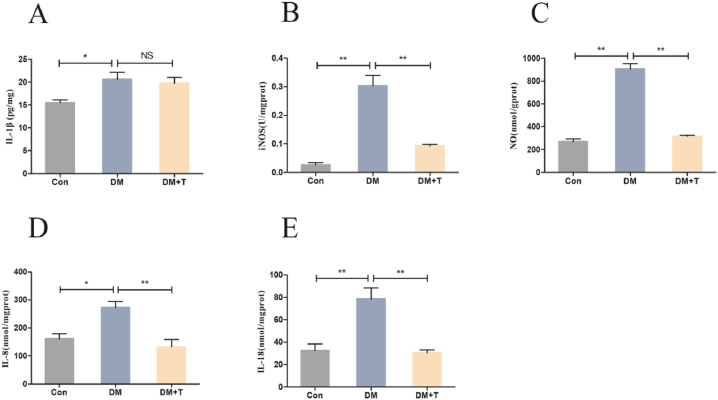
Results of liver proinflammatory factor assay of T2DM rats**.** (**A**) The liver IL-1β levels of T2DM rats. (**B**) The liver iNOS levels of T2DM rats. (**C**) The liver NO levels of T2DM rats. (**D**) The IL-8 levels of T2DM rats. (**E**) The IL-18 levels of T2DM rats. The results were expressed by the mean ± SEM. *p < 0.05, **p < 0.01 and NS (p > 0.05).

In Fig. 5, the levels of expression of factors related to M1 KCs were examined. As shown in Fig. 5A–E, compared with the normal group, the levels of IL-1β, iNOS, NO, IL-8 and IL-18 of the diabetes model group were significantly increased (p < 0.05); the levels of IL-1β factors decreased following taurine administration, although there was no statistically significant difference (p > 0.05) between them and the levels of iNOS, NO, IL-8, and IL-18 factors were considerably decreased (p < 0.01).

### 3.6 Effect of taurine on the JAK2-STAT1 signalling pathway

**Fig. 6:**
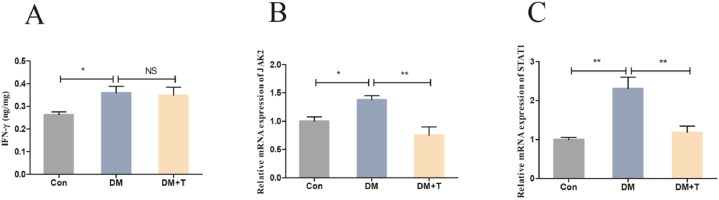
Results of the JAK2-STAT1 signalling pathway-related factor assay. (**A**) The liver IFN-γ levels of T2DM rats. (**B**) The JAK2 mRNA expression of T2DM rats. (**C**) The STAT1 mRNA expression of T2DM rats. The results were expressed by the mean ± SEM. *p < 0.05, **p < 0.01 and NS (p > 0.05).

The JAK2-STAT1 signalling pathway is an important pathway that regulates macrophage polarization. Therefore, we examined the factors expression which related to the JAK2-STAT1 signalling pathway, and the results were showed in Fig. 6A–C. IFN-γ content tended to decrease with taurine administration, but there was no statistically significant difference (p > 0.05) between the diabetic model group and the normal group in terms of IFN-γ levels (p < 0.05). The diabetes model group's JAK2 and STAT1 mRNA expression levels were found to be considerably higher than those in the normal group (p < 0.05), and taurine treatment dramatically decreased these levels (p < 0.01).

### 3.7 Effect of taurine on the TLR4-NF-κB signalling pathway

**Fig. 7:**
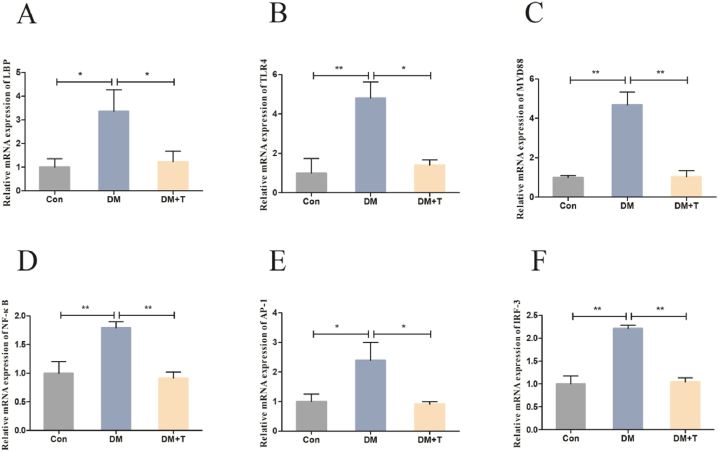
Results of the LPS-TLR4-NF-κB inflammatory signalling pathway assay. (**A**) The LBP mRNA expression of T2DM rats. (**B**) The TLR4 mRNA expression of T2DM rats. (**C**) The MyD88 mRNA expression of T2DM rats. (**D**) The NF-κB mRNA expression of T2DM rats. (**E**) The AP-1 mRNA expression of T2DM rats. (**F**) The IRF-3 mRNA expression of T2DM rats. The results were expressed by the mean ± SEM. *p < 0.05 and **p < 0.01.

We assessed the TLR4/NF-κB signaling pathway's activity since it is crucial for controlling inflammation. As shown in Fig. 7A–F. When compared to the normal group, the diabetes model group's mRNA expression levels for LBP, TLR4, MyD88, NF-κB, AP-1, and IRF-3 were significantly higher (p < 0.05); after taurine treatment, we discovered that these mRNA levels had decreased significantly (p < 0.05).

### 3.8 Effect of taurine on NLRP3 inflammatory vesicles

**Fig. 8:**
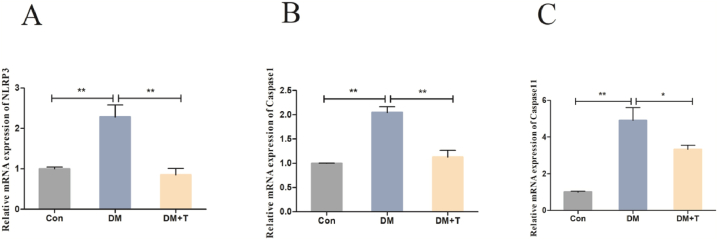
Results of the NLRP3 inflammatory vesicle-associated factor assay. (**A**) The NLRP3 mRNA expression of T2DM rats. (**B**) The Caspase1 mRNA expression of T2DM rats. (**C**) The Caspase11 mRNA expression of T2DM rats. The results were expressed by the mean ± SEM. *p < 0.05 and **p < 0.01.

Then, we investigated if the suppression of NLRP3 inflammatory vesicles was associated with taurine's amelioration of T2DM liver injury. Fig. 8A–C displays the findings from our analysis of the components whose expression levels were associated with NLRP3 inflammatory activity. The mRNA expression levels of NLRP3, Caspase-1, and Caspase-11 were significantly higher in the diabetic model group compared to the control group (p < 0.01); however, after taurine treatment, they were significantly lower (p < 0.05).

## 4 Discussion

With the improvements in living standards, the incidence of T2DM has increased significantly. T2DM is a complex metabolic disorder that is related to changes in lipid metabolism and pancreatic β-cell dysfunction caused by obesity, leading to insufficient insulin secretion and IR [25]. A major complication of T2DM is liver injury. Metabolic disorders associated with T2DM, such as hyperglycemia, IR, oxidative stress and inflammation, will cause liver injury, which eventually leads to a series of liver diseases, including fatty liver, cirrhosis and liver cancer [26,27]. In the present, there is no effective treatment for liver injury caused by type 2 diabetes. Therefore, exploring a safe and effective method to prevent and treat liver injury caused by T2DM is necessary. A large number of studies have reported that taurine has a good preventive and therapeutic effect on T2DM [18,19,22]. In order to investigate the influence of taurine on liver injury caused by T2DM, 8-week-old male SD rats were established a model of T2DM induced by sustained high-fat diets and intraperitoneal STZ injections (30 mg/kg). According to the experimental results, the FBG of T2DM rats was higher than 11.1 mmol/L three times and for more than two weeks, based on the above results, it is proved that the T2DM rat model is successfully established [28,29]. Histopathological observation of the liver showed that in the normal group, the structures of the liver cells were clear, and there was no inflammatory cell aggregation around the liver vessels; the morphological structures of the liver cells of the T2DM rats were disordered, and there were many inflammatory cells around the liver vessels of the T2DM rats. The examination of liver function indices showed that compared with the normal group, ALT and AST levels of T2DM rats were significantly increased, indicating the presence of liver injury in T2DM rats. Furthermore, we adopted taurine as a treatment and found that the taurine treatment (DM + T) group had significantly fewer liver lesions than the DM group, and the liver function index levels of the taurine treatment (DM + T) group were significantly decreased. Our study suggest that taurine could be beneficial for treating T2DM-induced liver injury.

Oxidative stress and inflammation are important factors affecting the occurrence and development of T2DM. Oxidative stress refers to the excessive production level of ROS and RNS; when the production level exceeds the normal physiological range, it leads to the aggregation of oxidation products, and thus, the cell structure is destructed. When T2DM occurs, hyperglycemia leads to nonenzymatic glycosylation of cells and tissues, resulting in a large amount of ROS produced and leading to oxidative stress in the liver; furthermore, obesity caused by a high-fat diet is typically associated with increased liver oxidative stress. As important indicators of oxidative stress, SOD, GSH-Px and the lipid peroxidation product MDA are typically used to evaluate the level of oxidative stress [30]. When oxidative stress occurs, SOD, CAT and GSH-Px in the liver are inhibited, the T-AOC of the body decreases, and the production of the oxidative stress biomarker MDA increases. The antioxidant indices SOD, CAT, GSH-Px, and T-AOC were significantly reduced in T2DM rats, and the content of the lipid peroxidation product MDA was significantly increased. Our experimental results are similar with the research results of Liao Z, Liu H and other researchers [31]. After taurine treatment, antioxidant-related indicators were significantly increased(for example SOD, CAT, GSH-Px and T-AOC), and the content of the MDA was significantly decreased, indicating that taurine inhibited the occurrence of oxidative stress in the liver and improved the antioxidant capacity of the liver [32]. The research results of Fukuno S and others showed that The use of 10 mg/kg taurine can significantly increase the levels of SOD and GSH in the mouse liver injury model induced by 5-fluorouracil and inhibit the occurrence of oxidative stress in the liver [33]. Combined with our results, taurine can improve the antioxidant capacity of the liver.

Although we found that taurine could improve the antioxidant capacity of the liver, the specific mechanism is still unclear, and so we performed further experiments. Studies have shown that Nrf2 is a defence mechanism of the body or cells against internal and external stimuli. Nrf2 can sense the state of oxidative stress in cells and regulate the redox balance of cells, and its activation reduces the damage caused by oxidative stress. Nrf2 regulates a series of downstream genes, including HO-1, NQO1 and γ-GCS, inducing the production of antioxidant enzymes by binding to antioxidant response elements in promoter regions. Studies have shown that taurine supplementation in the diet can increase GSH-Px activity, enhance GSH synthesis, and prevent lipid peroxidation by regulating the Nrf2 signalling pathway in the liver inflammation model of broilers induced by lipopolysaccharide, thereby improving the antioxidant capacity of broilers. In addition, taurine increases HT22 cell resistance to glutamate-induced oxidative damage through p38/Nrf2-dependent HO-1 expression [34,35]. Therefore, we studied the effect of taurine on the expression of Nrf2 signalling pathway-related factors, and the experimental results were consistent with those of Liao Z, Guo J and others [31,36]. Our experimental results showed that the mRNA expression levels of Nrf2, HO-1, NQO1 and γ-GCS in the livers of T2DM rats were significantly decreased, and after treatment with taurine, the mRNA expression levels of Nrf2, HO-1, NQO1 and γ-GCS of rat livers were significantly increased. On the basis of these findings, we speculate that taurine will promote the expression of genes downstream of the Nrf2 antioxidant signalling pathway and the production of antioxidant enzymes by activating the Nrf2 antioxidant signalling pathway to improve the antioxidant capacity of the liver [36], reduce liver oxidative stress, and thus alleviate liver injury of T2DM rats.

Further enhancement of liver oxidative stress and the increase of oxidative stress products can activate liver macrophages to secrete lots of inflammatory cytokines and induce an inflammatory response, thus exacerbating T2DM liver injury [37,38]. Liver inflammation is mainly regulated by resident liver macrophages known as KCs. The surface marker of KCs is mucin-like hormone-like receptor 1 (F4/80), which recognizes exogenous and endogenous signals by expressing pattern recognition receptors (PRRs), such as Toll-like receptors (TLRs), nod-like receptors (NLRs) and mannose receptors. When stimulated by LPS and IFN-γ, KCs are polarized into the M1 type, of which the marker is scavenger receptor CD68, a large number of proinflammatory cytokines were released. We found that the mRNA expression levels of the M1-type KCs markers CD68 and CD68/(F4/80) of T2DM rats were significantly increased. This is consistent with the results of Hou B and others. In the HFD-STZ-induced T2DM rat model, the immunohistochemical results of liver CD68 showed that the infiltration of liver M1 macrophages was significantly increased [39]. After taurine treatment, M1-type KCs markers CD68 and CD68/(F4/80) mRNA expression levels were significantly reduced. These results show that taurine can reduce the polarization level of M1-type KCs. When KCs are polarized into the M1 type, they can promote the production of a variety of inflammatory factors, such as IL-1β, IL-8, IL-18 and IFN-γ [40]. Cytokines IL-1β and IFN-γ can upregulate iNOS expression. In addition, Nitric oxide (NO) can be produced by M1-type KCs, and excessive levels can cause inflammation and oxidative stress [41]. We found that the levels of liver inflammatory factors IL-1β, iNOS, NO, IL-8 and IL-18 in T2DM rats were significantly increased, indicating that the liver inflammatory response was aggravated [40]. Studies have shown that in the liver of T2DM rats, the expression levels of some cytokines (such as IL-1β, iNOS) were significantly increased. [42,43], and serum IL-8 and IL-18 levels were significantly increased in T2DM patients [44]. The results we observed are consistent with this: after treatment with taurine, IL-1β content was shown as a downward trend, but there is no statistical difference.; the contents of iNOS, NO, IL-8 and IL-18 were significantly decreased, indicating that taurine could reduce the expression of inflammatory factors in the liver and alleviate the inflammatory response of the liver [45]. Zheng J and others found that taurine supplementation could reduce the protein expression of IL-1β, IL-6 and TNF-α and alleviate liver inflammation in rat and mouse liver injury models [46,47]. Combined with our experimental results, it is speculated that taurine alleviates liver injury of T2DM rats by inhibiting the inflammatory response mediated by M1 macrophage polarization.

JAK2 is an important factor in regulating macrophage function, and in the macrophage polarization, the JAK2-STAT1 signalling pathway is important. In M1-type KCs, LPS or IFN-γ stimulation can activate JAK2, further activating the expression of STAT1, promoting the occurrence of the inflammatory response, and leading to liver injury [48]. We found that the content of IFN-γ in the T2DM rats’ livers was significantly increased, and the mRNA expression levels of JAK2 and STAT1 were significantly increased compared with the normal group. After taurine treatment, the content of IFN-γ was shown as a downward trend, but there was no significant difference, while the mRNA expression levels of JAK2 and STAT1 were significantly decreased. Lee and others' study showed that it can alleviate the inflammatory response of LPS-stimulated macrophages by inhibiting the JAK2-STAT1 signalling pathway [49]. Therefore, we believe that taurine can reduce the polarization level of M1-type KCs and reduce the release of proinflammatory cytokines by interdicting the JAK2-STAT1 signalling pathway, thereby reducing liver inflammation.

When T2DM develops, serum endotoxin (LPS) levels increase [50]. LPS binds to plasma LPS binding protein (LBP) and TLR4 to form a complex, which triggers the inflammatory cascade through the NF-κB pathway, activates the expression of transcription factors such as protein-1 (AP-1) and interferon regulatory factor-3 (IRF-3), promotes liver inflammatory factors increase (including TNF-α, IL-6 and IL-1β), and ultimately leads to liver injury [51]. Compared with normal rats, we found significant differences in mRNA expression of LBP, TLR4, MyD88, NF–B, AP-1 and IRF-3 in their livers. After taurine treatment, it was found that mRNA expression of LBP, TLR4, MyD88, NF-κB, AP-1, and IRF-3 were significantly decreased [52]. A study has shown that taurine inhibits TLR4/MyD88 signaling in chronically alcohol-fed rats to reduce liver inflammation [53]. It can be concluded that taurine inhibits the LPS-TLR4-NF-κB signalling pathway to reduce liver inflammation in rats with T2DM.

In the development of diabetes and its complications, the NLRP3 inflammasome plays an important role [54]. Islet β-cell failure of T2DM rats is related to the infiltration of M1 macrophages into islet cells. The NLRP3 inflammasome was activated in macrophages as a result of this process [55], which releases proinflammatory cytokines and mediates the occurrence of the inflammatory response. NLRP3 belongs to the NLR family and is a natural immune cell receptor. The NLRP3 inflammasome consists of NLRP3, apoptosis-related spot-like protein (ASC) and caspase-1. Studies have shown that the NLRP3 protein is expressed in macrophages, especially in M1 macrophages [56]. Activated of the NLRP3 inflammasome can occur through two pathways (the typical pathway or atypical pathway). The typical pathway includes two steps: priming and protein complex assembly [57]. The first step is to activate the NF-κB signalling pathway through innate immune signals of cytokine receptors (such as TNF-α and/or TLR4-MyD88), that leads to the activation of NLRP3, pro-caspase-1, and pro-IL-1β, then followed by the oligomerization of NLRP3 and ASC, resulting in the activation of caspase-1 and the release of IL-18 and IL-1β. In the atypical pathway, gram negative bacteria can trigger TLR4-MyD88 through NF-κB induction and increase the leves of IL-18, IL-1β, NLRP3, IRF-3, IRF-7 and other genes. IRF3-IRF7 complex induced the expression of IFN-α/β, so JAK/STAT pathway was activated and promoted the transcription of Caspase-11 gene [58,59]. Consistent with the results of Rai RC and others, our experimental results showed that the mRNA expression levels of NLRP3, Caspase-1 and Caspase-11 in the livers of T2DM rats were significantly increased compared with the normal group; after taurine treatment, those factors’ mRNA expression levels were significantly decreased [60], indicating that taurine could alleviate the inflammatory response of the liver by inhibiting the activation of NLRP3 inflammasome. The results of Qiu T and others showed that taurine supplementation could reduce the protein expression of NLRP3, ASC and Caspase-1 in liver tissue in an arsenic trioxide (AS2O3)-induced mouse hepatitis model [61]. Combined with our results, it is shown that taurine can inhibit NLRP3 inflammasome activated in the liver, and reduce the transcription level of inflammatory cytokines and the inflammatory response mediated by inflammatory cytokines, thereby inhibiting the liver inflammatory response of T2DM rats and alleviating liver injury.

## 5 Conclusion

The results of this study showed that taurine could alleviate liver injury of HFD/STZ-induced T2DM rats. The mechanism may involve improving the antioxidant capacity of the liver by activating the Nrf2 signalling pathway and its downstream antioxidant enzymes, the liver inflammatory response mediated by the JAK2/STAT1 and TLR4/NF-κB signalling pathways and inhibition of the NLRP3 inflammasome. These results provide new ideas for the application of taurine in the treatment or prevention of liver injury caused by T2DM.

## Funding

This work was supported by the 10.13039/501100001809National Natural Science Foundation of China (31972639, 32272695 and 31772694), Shenyang Science and Technology Department Plan Project (22-318-2-08) and the Scientific Research Funding Projects from the Science and Technology Department of Shenyang (RC220278).

## Data availability

All data generated or analysed during the present study are included in this published article. The data will be provided as required.

## CRediT authorship contribution statement

**Guangyi Ouyang:** Writing – original draft. **Nannan Wang:** Investigation. **Jihang Tong:** Validation. **Wenke Sun:** Investigation. **Jiancheng Yang:** Writing – review & editing, Writing – original draft, Supervision, Project administration, Funding acquisition, Data curation, Conceptualization. **Gaofeng Wu:** Supervision, Project administration, Funding acquisition, Conceptualization.

## Declaration of competing interest

The authors declare the following financial interests/personal relationships which may be considered as potential competing interests:Jiancheng Yang and Gaofeng Wu reports was provided by National Natural Science Foundation of China. Jiancheng Yang and Gaofeng Wu reports a relationship with 10.13039/501100001809National Natural Science Foundation of China that includes: funding grants. If there are other authors, they declare that they have no known competing financial interests or personal relationships that could have appeared to influence the work reported in this paper.

## References

[1] Cho N.H., Shaw J.E., Karuranga S., Huang Y., da Rocha Fernandes J.D., Ohlrogge A.W. (2018 Apr; (2018). IDF Diabetes Atlas: global estimates of diabetes prevalence for 2017 and projections for 2045. Diabetes Res. Clin. Pract..

[2] Hsiang J.C., Gane E.J., Bai W.W., Gerred S.J. (2015 Mar; (2015). Type 2 diabetes: a risk factor for liver mortality and complications in hepatitis B cirrhosis patients. J. Gastroenterol. Hepatol..

[3] Xia C., Zhang X., Cao T., Wang J., Li C., Yue L. (2020). Hepatic transcriptome analysis revealing the molecular pathogenesis of type 2 diabetes mellitus in zucker diabetic fatty rats. Front. Endocrinol..

[4] Wan Y., Garner J., Wu N., Phillip L., Han Y., McDaniel K. (2016 Feb; (2016). Role of stem cells during diabetic liver injury. J. Cell Mol. Med..

[5] Szkudelska K., Okulicz M., Hertig I., Szkudelski T. (2020 May; (2020). Resveratrol ameliorates inflammatory and oxidative stress in type 2 diabetic Goto-Kakizaki rats. Biomed. Pharmacother..

[6] Chen Z., Tian R., She Z., Cai J., Li H. (2020 May 20; (2020). Role of oxidative stress in the pathogenesis of nonalcoholic fatty liver disease. Free Radic. Biol. Med..

[7] Chen Z., Yu R., Xiong Y., Du F., Zhu S. (2017). A vicious circle between insulin resistance and inflammation in nonalcoholic fatty liver disease. Lipids Health Dis..

[8] Mohamed J., Nazratun Nafizah A.H., Zariyantey A.H., Budin S.B. (2016 May; (2016). Mechanisms of Diabetes-Induced Liver Damage: the role of oxidative stress and inflammation. Sultan. Qaboos Univ. Med. J..

[9] Farzanegi P., Dana A., Ebrahimpoor Z., Asadi M., Azarbayjani M.A. (2019 Aug; (2019). Mechanisms of beneficial effects of exercise training on non-alcoholic fatty liver disease (NAFLD): roles of oxidative stress and inflammation. Eur. J. Sport Sci..

[10] Friedman S.L., Neuschwander-Tetri B.A., Rinella M., Sanyal A.J. (2018 Jul; (2018). Mechanisms of NAFLD development and therapeutic strategies. Nat. Med..

[11] Arroyave-Ospina J.C., Wu Z., Geng Y., Moshage H. (2021). Role of oxidative stress in the pathogenesis of non-alcoholic fatty liver disease: implications for prevention and therapy. Antioxidants.

[12] Bkaily G., Jazzar A., Normand A., Simon Y., Al-Khoury J., Jacques D. (2020 Feb; (2020). Taurine and cardiac disease: state of the art and perspectives. Can. J. Physiol. Pharmacol..

[13] Wen C., Li F., Zhang L., Duan Y., Guo Q., Wang W. (2019). Taurine is involved in energy metabolism in muscles, adipose tissue, and the liver. Mol. Nutr. Food Res..

[14] Lambert I.H., Kristensen D.M., Holm J.B., Mortensen O.H. (2015 Jan; (2015). Physiological role of taurine--from organism to organelle. Acta Physiol..

[15] Imae M., Asano T., Murakami S. (2014 Jan; (2014). Potential role of taurine in the prevention of diabetes and metabolic syndrome. Amino Acids.

[16] Baliou S., Adamaki M., Ioannou P., Pappa A., Panayiotidis M.I., Spandidos D.A. (2021). Protective role of taurine against oxidative stress. Mol. Med. Rep..

[17] Maleki V., Mahdavi R., Hajizadeh-Sharafabad F., Alizadeh M. (2020). The effects of taurine supplementation on oxidative stress indices and inflammation biomarkers in patients with type 2 diabetes: a randomized, double-blind, placebo-controlled trial. Diabetol. Metab. Syndrome.

[18] Faghfouri A.H., Seyyed Shoura S.M., Fathollahi P., Shadbad M.A., Papi S., Ostadrahimi A. (2022). Profiling inflammatory and oxidative stress biomarkers following taurine supplementation: a systematic review and dose-response meta-analysis of controlled trials. Eur. J. Clin. Nutr..

[19] Murakami S., Funahashi K., Tamagawa N., Ning M., Ito T. (2022). Taurine ameliorates streptozotocin-induced diabetes by modulating hepatic glucose metabolism and oxidative stress in mice. Metabolites.

[20] Shi Y., Zhong L., Fan Y., Zhang J., Dai J., Zhong H. (2022). Taurine inhibits hydrogen peroxide-induced oxidative stress, inflammatory response and apoptosis in liver of Monopterus albus. Fish Shellfish Immunol..

[21] Abd-Elhakim Y.M., Ghoneim M.H., Ebraheim L.L.M., Imam T.S. (2020). Taurine and hesperidin rescues carbon tetrachloride-triggered testicular and kidney damage in rats via modulating oxidative stress and inflammation. Life Sci..

[22] Ma Y., Zhang Y., Li R., Deng S., Qin Q., Ran C. (2022). Mechanism of taurine reducing inflammation and organ injury in sepsis mice. Cell. Immunol..

[23] Liu Y., Li F., Zhang L., Wu J., Wang Y., Yu H. (2017). Taurine alleviates lipopolysaccharide-induced liver injury by anti-inflammation and antioxidants in rats. Mol. Med. Rep..

[24] Wu G., San J., Pang H., Du Y., Li W., Zhou X. (2022). Taurine attenuates AFB1-induced liver injury by alleviating oxidative stress and regulating mitochondria-mediated apoptosis. Toxicon.

[25] Podell B.K., Ackart D.F., Richardson M.A., DiLisio J.E., Pulford B., Basaraba R.J. (2017). A model of type 2 diabetes in the Guinea pig using sequential diet-induced glucose intolerance and streptozotocin treatment. Dis. Model Mech..

[26] Shima T., Uto H., Ueki K., Takamura T., Kohgo Y., Kawata S. (2013). Clinicopathological features of liver injury in patients with type 2 diabetes mellitus and comparative study of histologically proven nonalcoholic fatty liver diseases with or without type 2 diabetes mellitus. J. Gastroenterol..

[27] Bedi O., Aggarwal S., Trehanpati N., Ramakrishna G., Krishan P. (2019). Molecular and pathological events involved in the pathogenesis of diabetes-associated nonalcoholic fatty liver disease. J. Clin. Exp. Hepatol..

[28] Abdel-Rahman R.F., Ezzat S.M., Ogaly H.A., Abd-Elsalam R.M., Hessin A.F., Fekry M.I. (2020). Ficus deltoidea extract down-regulates protein tyrosine phosphatase 1B expression in a rat model of type 2 diabetes mellitus: a new insight into its antidiabetic mechanism. J. Nutr. Sci..

[29] Zepeda-Peña A.C., Gurrola-Díaz C.M., Domínguez-Rosales J.A., García-López P.M., Pizano-Andrade J.C., Hernández-Nazará Z.H. (2021). Effect of Lupinus rotundiflorus gamma conglutin treatment on JNK1 gene expression and protein activation in a rat model of type 2 diabetes. Pharm. Biol..

[30] Wang G., Wu B., Zhang L., Jin X., Wang K., Xu W. (2021). The protective effects of trelagliptin on high-fat diet-induced nonalcoholic fatty liver disease in mice. J. Biochem. Mol. Toxicol..

[31] Liao Z., Zhang J., Liu B., Yan T., Xu F., Xiao F. (2019). Polysaccharide from okra (*Abelmoschus esculentus*(L.) moench) improves antioxidant capacity via PI3K/AKT pathways and Nrf2 translocation in a type 2 diabetes model. Molecules.

[32] Liu H., Li N., Jin M., Miao X., Zhang X., Zhong W. (2020 Aug; (2020). Magnesium supplementation enhances insulin sensitivity and decreases insulin resistance in diabetic rats. Iran J. Basic Med. Sci..

[33] Fukuno S., Nagai K., Yoshida S., Suzuki H., Konishi H. (2016). Taurine as a protective agent for 5-fluorouracil-induced hepatic damage related to oxidative stress. Pharmazie.

[34] Han H., Zhang J., Chen Y., Shen M., Yan E., Wei C. (2020). Dietary taurine supplementation attenuates lipopolysaccharide-induced inflammatory responses and oxidative stress of broiler chickens at an early age. J. Anim. Sci..

[35] Lee D.S., Cheong S.H. (2017). Taurine have neuroprotective activity against oxidative damage-induced HT22 cell death through heme oxygenase-1 pathway. Adv. Exp. Med. Biol..

[36] Guo J., Li J., Wei H., Liang Z. (2021). Maackiain protects the kidneys of type 2 diabetic rats via modulating the Nrf2/HO-1 and TLR4/NF-κB/Caspase-3 pathways. Drug Des. Dev. Ther..

[37] Day C.P., James O.F. (1998). Steatohepatitis: a tale of two "hits". Gastroenterology.

[38] Kazankov K., Jørgensen S.M.D., Thomsen K.L., Møller H.J., Vilstrup H., George J. (2019). The role of macrophages in nonalcoholic fatty liver disease and nonalcoholic steatohepatitis. Nat. Rev. Gastroenterol. Hepatol..

[39] Hou B., Zhao Y., Qiang G., Yang X., Xu C., Chen X. (2018). Puerarin mitigates diabetic hepatic steatosis and fibrosis by inhibiting TGF-*β* signaling pathway activation in type 2 diabetic rats. Oxid. Med. Cell. Longev..

[40] Meniailo M.E., Malashchenko V.V., Shmarov V.A., Gazatova N.D., Melashchenko O.B., Goncharov A.G. (2018). Interleukin-8 favors pro-inflammatory activity of human monocytes/macrophages. Int. Immunopharm..

[41] Rath M., Müller I., Kropf P., Closs E.I., Munder M. (2014). Metabolism via arginase or nitric oxide synthase: two competing arginine pathways in macrophages. Front. Immunol..

[42] Yu S., Cheng Y., Zhang L., Yin Y., Xue J., Li B. (2019). Treatment with adipose tissue-derived mesenchymal stem cells exerts anti-diabetic effects, improves long-term complications, and attenuates inflammation in type 2 diabetic rats. Stem Cell Res. Ther..

[43] Cassano V., Leo A., Tallarico M., Nesci V., Cimellaro A., Fiorentino T.V. (2020). Metabolic and cognitive effects of ranolazine in type 2 diabetes mellitus: data from an in vivo model. Nutrients.

[44] Cimini F.A., Barchetta I., Porzia A., Mainiero F., Costantino C., Bertoccini L. (2017). Circulating IL-8 levels are increased in patients with type 2 diabetes and associated with worse inflammatory and cardiometabolic profile. Acta Diabetol..

[45] Talebian R., Hashem O., Gruber R. (2020). Taurocholic acid lowers the inflammatory response of gingival fibroblasts, epithelial cells, and macrophages. J. Oral Sci..

[46] Zheng J., Qiu G., Zhou Y., Ma K., Cui S. (2023). Hepatoprotective effects of taurine against cadmium-induced liver injury in female mice. Biol. Trace Elem. Res..

[47] Beigi T., Safi A., Satvati M., Kalantari-Hesari A., Ahmadi R., Meshkibaf M.H. (2022). Protective role of ellagic acid and taurine against fluoxetine induced hepatotoxic effects on biochemical and oxidative stress parameters, histopathological changes, and gene expressions of IL-1β, NF-κB, and TNF-α in male Wistar rats. Life Sci..

[48] Liu P., Li H., Gong J., Geng Y., Jiang M., Xu H. (2022). Chitooligosaccharides alleviate hepatic fibrosis by regulating the polarization of M1 and M2 macrophages. Food Funct..

[49] Lee N., Heo Y.J., Choi S.E., Jeon J.Y., Han S.J., Kim D.J. (2021). Anti-inflammatory effects of empagliflozin and gemigliptin on LPS-stimulated macrophage via the IKK/NF-*κ*B, MKK7/JNK, and JAK2/STAT1 signalling pathways. J. Immunol. Res..

[50] Zeng Z., Guo X., Zhang J., Yuan Q., Chen S. (2021). *Lactobacillus paracasei* modulates the gut microbiota and improves inflammation in type 2 diabetic rats. Food Funct..

[51] Jialal I., Kaur H., Devaraj S. (2014). Toll-like receptor status in obesity and metabolic syndrome: a translational perspective. J. Clin. Endocrinol. Metab..

[52] Lin C.J., Chiu C.C., Chen Y.C., Chen M.L., Hsu T.C., Tzang B.S. (2015). Taurine attenuates hepatic inflammation in chronic alcohol-fed rats through inhibition of TLR4/MyD88 signaling. J. Med. Food.

[53] Wu G., Yang Q., Yu Y., Lin S., Feng Y., Lv Q. (2017). Taurine inhibits kupffer cells activation induced by lipopolysaccharide in alcoholic liver damaged rats. Adv. Exp. Med. Biol..

[54] Dixit V.D. (2013). Nlrp3 inflammasome activation in type 2 diabetes: is it clinically relevant?. Diabetes.

[55] Jourdan T., Godlewski G., Cinar R., Bertola A., Szanda G., Liu J. (2013). Activation of the Nlrp3 inflammasome in infiltrating macrophages by endocannabinoids mediates beta cell loss in type 2 diabetes. Nat. Med..

[56] Awad F., Assrawi E., Jumeau C., Georgin-Lavialle S., Cobret L., Duquesnoy P. (2017). Impact of human monocyte and macrophage polarization on NLR expression and NLRP3 inflammasome activation. PLoS One.

[57] Paik S., Kim J.K., Silwal P., Sasakawa C., Jo E.K. (2021). An update on the regulatory mechanisms of NLRP3 inflammasome activation. Cell. Mol. Immunol..

[58] Pellegrini C., Antonioli L., Lopez-Castejon G., Blandizzi C., Fornai M. (2017). Canonical and non-canonical activation of NLRP3 inflammasome at the crossroad between immune tolerance and intestinal inflammation. Front. Immunol..

[59] Rathinam V.A., Fitzgerald K.A. (2016). Inflammasome complexes: emerging mechanisms and effector functions. Cell.

[60] Rai R.C., Bagul P.K., Banerjee S.K. (2020). NLRP3 inflammasome drives inflammation in high fructose fed diabetic rat liver: effect of resveratrol and metformin. Life Sci..

[61] Qiu T., Pei P., Yao X., Jiang L., Wei S., Wang Z. (2018). Taurine attenuates arsenic-induced pyroptosis and nonalcoholicsteatohepatitis by inhibiting the autophagic-inflammasomal pathway. Cell Death Dis..

